# The risk factors of vertebral refracture after kyphoplasty in patients with osteoporotic vertebral compression fractures: a study protocol for a prospective cohort study

**DOI:** 10.1186/s12891-018-2123-6

**Published:** 2018-07-02

**Authors:** Lei Feng, Chun Feng, Jie Chen, Yu Wu, Jin-Ming Shen

**Affiliations:** 1 Department of Orthopaedics, The First Affiliated Hospital of Zhejiang Chinese Medicine University, 9 Ninth Avenue, Hangzhou Economic and Technological Development Zone, Zhejiang, 310018 Hangzhou China; 2 The Second Affiliated Hospital of Zhejiang University School of Medicine, Zhejiang, 310006 Hangzhou China

**Keywords:** Osteoporotic vertebral compression fracture (OVCF), Kyphoplasty, Refracture, Bone mineral density (BMD)

## Abstract

**Background:**

Percutaneous kyphoplasty (PKP) is the first-line treatment for osteoporotic vertebral compression fractures (OVCFs) that can immediately relieve pain and allow the quick recovery of lost mobility. However, some studies reported that after PKP, the incidence of vertebral refracture, particularly adjacent vertebral fracture (AVF), was high. Our previous meta-analysis suggested that the risks for vertebral refracture and AVF did not increase after percutaneous vertebral augmentation in OVCF patients. Despite the negative results of our meta-analysis, there is still significant evidence regarding the relationship between kyphoplasty and AVF, so a new prospective cohort study is warranted. In addition, in our previous retrospective study, we found that advanced age, female sex and low oestradiol (E_2_) concentrations might be related to the occurrence of postoperative vertebral refracture after PKP. To sufficiently evaluate the probable factors involved in the occurrence of postoperative vertebral refracture, we designed this prospective study.

**Methods:**

This is a prospective cohort study of patients admitted for PKP to treat painful OVCFs. The baseline data, including demographic information, lifestyle, bone metabolic status, sex hormone and sex hormone-binding globulin (SHBG) levels, and clinical characteristics will be collected at the time of enrolment. Surgical features of PKP will be recorded on the operation day. Lifestyle, bone metabolic status, sex hormone levels, and SHBG levels will be assessed during the follow-up period at 1 m, 3 m, 12 m, and 24 m postoperatively. Patients suffering from acutely aggravated back pain will be referred to an orthopaedist, and refractured vertebrae will be confirmed by magnetic resonance imaging and computed tomography. The primary outcome will be the incidence of vertebral refracture. Multivariate analyses will be carried out to evaluate the variables that are independently correlated with vertebral refracture.

**Discussion:**

To evaluate the risk of postoperative refracture preoperatively and to identify the surgical points related to postoperative refracture, this study will explore the risk factors related to vertebral refracture after PKP. The results may provide new information about defining OVCF patients suitable for PKP treatment.

**Trial registration:**

ChiCTR-ROC-17011562. Registered on July 4th, 2017.

## Background

Every year, there are approximately 1,110,000 people in China [1] and 700,000 people in the United States [2] who suffer from osteoporotic vertebral compression fractures (OVCFs). Therefore, the treatment of these patients is an important topic to improve patients’ quality of life and reduce the burden on society. The first-line therapy for OVCF patients is conservative treatment, including medication, orthotic bracing, and physical therapy. In recent years, percutaneous kyphoplasty (PKP) has become more prevalent worldwide because it can rapidly relieve pain and dramatically improve patients’ quality of life.

A series of studies have compared the therapeutic effects between conservative treatment and percutaneous vertebral augmentation. In 2009, a multicenter randomized controlled trial (RCT) compared 68 OVCF patients who underwent vertebroplasties and 63 patients who underwent simulated procedures (control group) and found that improvements in pain and pain-related disability were similar between the two groups [3]. However, in 2011, an RCT performed in Belgium that compared 149 cases treated with kyphoplasty and 151 cases treated with conservative therapy found that kyphoplasty rapidly reduced pain and improved function, disability, and quality of life, without increasing the risk of additional vertebral fractures [4]. In 2013, a multicenter RCT performed in Europe compared kyphoplasty and conservative therapy for the treatment of acute painful vertebral fractures and found that kyphoplasty achieved pain relief and functional recovery and rapidly improved patients’ quality of life [5]. In 2016, a multicenter double-blind RCT performed in Australia indicated that in patients with acute OVCF, vertebroplasty was superior to placebo for pain relief [6]. A recent meta-analysis involving ten RCTs indicated that compared with conservative therapy, vertebroplasty/kyphoplasty was associated with greater pain relief, significant improvement in daily function, and higher quality of life [7]. The majority of studies support the finding that vertebroplasty/kyphoplasty is beneficial for acute OVCF.

However, some reports suggested that the risk of new vertebral fractures might be elevated after percutaneous vertebral augmentation. More than ten years ago, a retrospective analysis performed in the USA suggested that after vertebroplasty the risk of new vertebral fractures increased, particularly adjacent level fractures, and found that they occurred earlier than non-adjacent level fractures [8]. However, the subsequent studies reported inconsistent results and did not support the correlation between PKP and vertebral refracture [9]. There have been several RCT studies investigating the risk of new vertebral fractures after percutaneous vertebral augmentation in patients of OVCF all over the world, including in China [10–13], Iran [14], Australia [15–17], the Netherlands [18, 19], Spain [20], and Denmark [21]. We reviewed these studies and performed a meta-analysis, which suggested no increase in the risk of new vertebral fracture or adjacent vertebral fracture [22].

If the high incidence of vertebral refracture is not associated with PKP, it is possible that underlying osteoporosis is the leading factor that results in the onset of vertebral fracture rather than percutaneous vertebral augmentation. Advanced age and decreased bone mineral density (BMD) scores were found to be correlated with the risk of AVF following PVP [23]. We carried out a retrospective study and found that advanced age, female sex and low oestradiol (E_2_) concentrations might be risk factors associated with postoperative vertebral refracture after kyphoplasty [24].

To more clearly and sufficiently evaluate the probable factors involved in the occurrence of postoperative vertebral refracture, we designed this prospective study to explore these risk factors. According to published reports and our previous study, we focused on the following aspects. 1) General patient characteristics may be important because advanced age is generally recognized as a risk factor for new vertebral fracture after vertebral augmentation, but it is unclear whether there should be an age limit for PKP treatment and what the cutoff age is [23–25]. 2) Bone metabolic status, which is reflected by BMD, serum bone metabolic markers, and the effect of anti-osteoporotic treatments, may be one of the dominant risk factors for refracture [26, 27]. 3) Sex hormone levels may be important, because numerous studies reported the association between sex hormone binding globulin (SHBG) and new or worsening vertebral fracture, although no association with E_2_ was found, indicating a role of SHBG in the pathogenesis of postoperative vertebral refracture [28–30]. Our previous study found that low E_2_ concentration might be associated with vertebral refracture after kyphoplasty [24]. 4) Some surgical factors, including balloon volume, cement volume, recovery of vertebral height, and cement leakage may be correlated with the incidence of vertebral fracture after kyphoplasty [26, 31]. 5) Lifestyle factors may also be involved. A study in the United States found that male smokers had a significant risk of low volumetric BMD and vertebral fractures [32]. Tobacco consumption may also be an independent predictor of non-vertebral fracture [33]. Although previous studies showed that these factors may be involved in fracture recurrence, more studies are required to further explore the role of these different factors. This prospective cohort study is designed to investigate the factors involved in the occurrence of postoperative refracture, which may provide some useful information for defining the target population for kyphoplasty and improving surgical techniques. If this study can be carried out successfully, it may be helpful for reducing surgical complications such as the occurrence of new vertebral fractures.

### Primary objectives

- To explore the possible factors involved in the occurrence of postoperative refracture, such as general characteristics, bone metabolic status, anti-osteoporotic treatment, sex hormone and SHBG levels, and the PKP procedures.
- To clarify and define the patients with OVCF suitable for PKP treatment with a low risk of postoperative refracture.

## Methods

### Study design

**Fig. 1:**
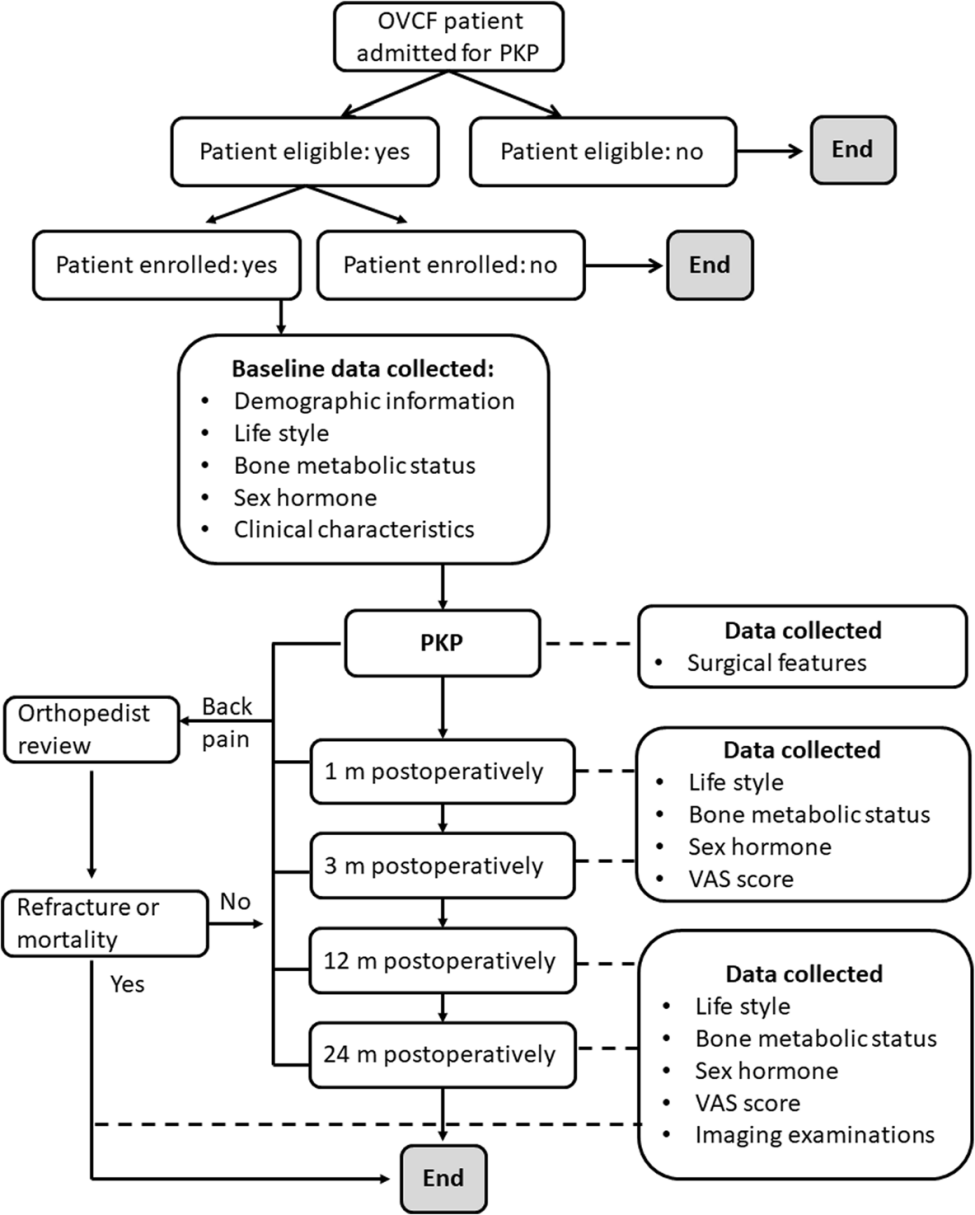
Study design

The present study is a prospective cohort study. Participant recruitment for the study will begin in Apr 2018 and continue until Mar 2019. As shown in Fig. 1, patients with OVCF who are admitted to the First Affiliated Hospital of Zhejiang Chinese Medicine University will be assessed by an orthopaedist to ensure that they are eligible for the study and will sign an informed consent form and be enrolled in this study. All the candidate patients will be recorded in a log that contains the demographic data and the reasons for non-participation in the study. The study protocol has been approved by the Ethics Committee of the First Affiliated Hospital of Zhejiang Chinese Medicine University.

### Study setting

This study will be carried out at the First Affiliated Hospital of Zhejiang Chinese Medicine University, which is a tertiary care centre that integrates medical treatment, medical education, scientific research, rehabilitation and healthcare. The hospital occupies 6.13 ha of land with 102,000 square metres of floor space for medical service and has 1500 inpatient beds. The department of orthopaedics is located at two sites, including the Hubin Campus and the Xiasha Campus, and has a total of 160 inpatient beds. All the patients will be recruited when they are admitted to the hospital to undergo PKP to treat painful OVCFs.

### Inclusion and exclusion criteria

The inclusion criteria are as follows: acute OVCFs of the thoracic, thoracolumbar, or lumbar spine with symptom duration of less than three weeks. An osteoporotic fracture is defined as a fracture that occurs spontaneously or secondary to mild trauma during daily activities. A VCF is diagnosed based on radiographs and computed tomography (CT) scans. Magnetic resonance imaging (MRI) examinations are carried out to confirm new OVCFs when patients are experiencing marked pain.

The exclusion criteria are as follows: A. previous vertebral fracture; B. previous major spinal surgery; C. complications involving malignant tumours; and D. fractures secondary to high-energy trauma, such as falls from high places.

### Sample size

The sample size calculation was performed using Epi Info 7.2 software (www.cdc.gov/epiinfo/index.html) for the following risk factors: age and E_2_ level. Based on our previous retrospective study, the ratio of patients < 80 years old and those ≥ 80 years old is nearly 5:1, and the ratio of patients with E_2_ levels ≥ 50 pmol/L and those < 50 pmol/L is nearly 3:1 [24]. A two-sided test will be set with an α risk of 0.05 and a power of 80%. Our previous retrospective study showed that the incidence of refracture was nearly 9% in the patients < 80 years old, 33% in patients ≥ 80 years old, 2% in patients with E_2_ levels ≥ 50 pmol/L, and 40% in patients with E_2_ levels < 50 pmol/L. The calculated required sample size for the cohort is 164 patients to evaluate the risk factor of age and 50 patients to evaluate the risk factor of E_2_ levels. Therefore, assuming a rate of loss to follow-up of 10%, 180 participants will be included.

### Data collection

As shown in Fig. 1, when a patient is enrolled in this study, the baseline data will be collected, including the demographic information, lifestyle, bone metabolic status, sex hormone levels, and clinical characteristics. The surgical features will be recorded during PKP. The assessment of lifestyle, bone metabolic status, sex hormone levels, SHBG levels, and clinical characteristics will be repeated at 1 m, 3 m, 12 m, and 24 m postoperatively. Complications will be defined by the Post-Operative Morbidity Survey and recorded [34]. Those who develop aggravated back pain will be reviewed by an orthopaedist, and the information regarding lifestyle, bone metabolic status, sex hormone levels, SHBG levels, and imaging examinations will be collected if refracture or mortality is confirmed. All the data will be recorded by two researchers to ensure the accuracy of data.

### Anthropometric data

The baseline data, including gender, age, weight, height, and BMI will be collected following participant recruitment (Table 1). Body weight and height will be measured twice with a height and weight scale (HW-900B, Kaiyuan Electronic Co., Zhengzhou, China) while the patients are wearing light clothes and are barefoot, and then the mean reading will be recorded. Body mass index (BMI) will be calculated as the body weight in kilograms divided by the square of the body height in metres.

**Table 1:** Timeline of data collection

Data collection		Pre	Op	1 m	3 m	12 m	24 m	OR
Demographic information	Gender	√						
	Age	√						
	Height	√						
	Weight	√						
	BMI	√						
Life style	Tobacco consumption	√		√	√	√	√	√
Bone metabolic status	BMD	√		√	√	√	√	√
	Bone metabolic markers	√		√	√	√	√	√
	Anti-osteoporosis treatment	√		√	√	√	√	√
Sex hormone	Serum hormone concentrations	√		√	√	√	√	√
	SHBG	√		√	√	√	√	√
Clinical characteristics	Affected vertebra number	√						
	VAS score	√		√	√	√	√	√
Surgical features	Balloon volume		√					
	Cement volume		√					
	Vertebral height	√	√			√	√	√
	Bone cement leakage		√					

Pre: pre-operation. Op: operation. 1 m, 3 m, 12 m, 24 m: one month, three months, 12 months, 24 months postoperatively. *OR* Orthopedist review

### Lifestyle

The patients’ use of cigarettes will be assessed upon admission to the hospital and monitored during follow-up at 1 m, 3 m, 12 m, and 24 m postoperatively.

To investigate smoking status, a questionnaire consisting of the following three questions will be used: (1) Have you ever smoked cigarettes regularly? (2) “Did you smoke prior to sustaining a vertebral compression fracture?” If yes, “How many cigarettes did you smoke each day on average?” If no, “How many months prior to sustaining a vertebral compression fracture did you stop smoking?” (3) “Have you smoked any cigarettes in the past seven days?” If yes, “How many cigarettes do you smoke each day on average?” If no, “When did you stopped smoking and why?” Then, the patients will be divided into never smokers, ex-smokers, current smokers, and heavy smokers.

### Bone mineral density

BMD scores of the lower lumbar spine (L2-L4) and unilateral hip (total hip) will be measured using dual-energy X-ray absorptiometry (DXA) (Hologic, Waltham, MA, USA). Blinded professional trained technicians will perform the DXA scans. BMD will be expressed in g/cm^2^ with T-scores (standard deviation (SD) differing from the mean BMD of young women). Osteoporosis will be defined based on the region with the lowest BMD and classified according to the World Health Organization (WHO) classification. A T-score > − 1.0 SD is defined as normal; a T-score ranging from − 1.0 SD to − 2.5 SD is defined as osteopenic; and a T-score ≤ -2.5 SD is defined as osteoporotic.

### Bone metabolic markers

Serum bone alkaline phosphatase (BAP) will be used as a marker of bone formation. BAP levels will be measured with an enzyme immunoassay (EIA) method (Sumitomo Biomedical, Osaka, Japan).

Urinary N-terminal cross-linking telopeptide of type I collagen (NTX) will be used as a marker of bone resorption. NTX levels will be examined with an enzyme-linked immunosorbent assay (ELISA) method (OSTEOMARK; Osteox International, Seattle, WA).

### Number of affected vertebra

The number of affected vertebra will be assessed via preoperative imaging examinations, including radiographs, CT, and MRI.

### Serum sex hormone concentration

Serum levels of follicle-stimulating hormone (FSH), luteinizing hormone (LH), testosterone (TE), E_2_, and SHBG will be measured by electrochemiluminescence immunoassays (ECLIA) using a Roche Modular E170 immunoassay analyser (Roche Diagnostics, Indianapolis, IN, USA).

### VAS score

The visual analogue scale (VAS) score will be assessed by a blinded trained nurse to estimate pain perception when the patient is admitted to the hospital and at 1 m, 3 m, 12 m, and 24 m after PKP [35]. The standard scale from 0 (no pain) to 10 (intolerable pain) will be adopted for pain analysis.

### Anti-osteoporotic treatment

Zoledronic acid is an anti-osteoporosis agent that rapidly and markedly increases the BMD of vertebral bone. Injections of zoledronic acid (Aclasta, Novartis Pharma Schweiz AG) will be recommended for patients who do not have contraindications for receiving zoledronic acid. If the patients agree to receive zoledronic acid treatment, 5 mg of Aclasta (Novartis Pharma Schweiz AG) will be administered 3 days after PKP, and Vitamin D and calcium supplements will also be continued.

### PKP features

Bipedicular kyphoplasty will be performed in this study. The balloon volume (BV), cement volume (CV), recovery of vertebral height, and bone cement leakage will be evaluated as factors that influence the risk of vertebral refracture. 1) The balloon volume will be recorded by the surgeons during the operation. 2) The cement volume will be recorded by the surgeons during the operation. 3) The anterior height of the fractured vertebra will be immediately measured with the picture archiving and communication systems (PACS) imaging display software (Zhejiang Public Information Industry Co., China) when the patients are enrolled and at 12 m and 24 m after PKP. Recovery of vertebral height will be defined as the anterior height of the fractured vertebra/the average height of the two adjacent vertebrae. 4) Bone cement leakage will be evaluated using radiographic evaluation during operation.

### Analysis

Quantitative variables will be summarized by the mean ± SD, and qualitative variables will be presented as counts and percentages. Continuous parameters of patients with and without vertebral refracture will be compared by analysis of variance (ANOVA), and binary parameters will be evaluated with logistic regression. A Pearson regression model will be used to analyse the correlation between the incidence of vertebral refracture and the covariates. Univariate and multivariate logistic regression analyses will be carried out to identify the independent predictors of vertebral refracture. A value of *P* < 0.05 will be considered statistically significant. Statistical analyses will be carried out using the SPSS 19.0 statistics package (SPSS, Chicago, IL, USA).

### Amendment of the protocol

A formal amendment to the protocol will be required before any modifications to the protocol are made. Any amendments will be approved by the Ethics Committee of the First Affiliated Hospital of Zhejiang Chinese Medicine University before implementation.

### Confidentiality

Every participant will be coded by a unique number when they are included in this study. The unique number will be used to identify the participant throughout the study. The pairing between the participant and the identity number will be password protected.

### Ethics and dissemination

This study has been approved by the Ethics Committee of the First Affiliated Hospital of Zhejiang Chinese Medicine University. This study has been registered online (http://www.chictr.org.cn/index.aspx) with the identifier ChiCTR-ROC-17011562 (registered on July 4th, 2017). The participants will be asked to sign an informed consent form before they are included in the study. The principles of the Declaration of Helsinki [36] and the WHO standards for observational studies [37] will be followed throughout the study.

A variety of methods will be adopted to maximize the visibility of the present study. First, we will try to publish this study protocol, which introduces the importance of evaluating the risk factors for vertebral refracture after PKP and describing the design. Second, to introduce the results, oral and poster presentations at national and international meetings will be encouraged to disseminate the findings. In addition, the study results will be published in a high-impact scientific journal to achieve maximum distribution.

We will deliver a deidentified dataset to the Clinical Trial Management Public Platform for release before December 2021.

## Discussion

This study will explore the following risk factors for vertebral refracture after PKP: 1) general characteristics, lifestyle, bone metabolic status, and sex hormone levels to identify the patients tend to suffer from refracture after PKP and to provide sufficient consultation for these patients and 2) surgical features to identify the key points of PKP that may help to reduce the occurrence postoperative refracture. The results may provide new information about the OVCF patients suitable for PKP treatment.

The limitations of the present study are as follows. The current guidelines for the treatment of OVCF recommend a combination treatment method including anti-osteoporotic treatment regardless of the major treatment method used to reduce the incidence of refractures. Therefore, we used combination treatment with Aclasta to treat OVCF. However, when the results of this study are compared with other populations, the rates of adjacent level fractures as well as the risk factors for fractures after PKP may be different than those of other populations, such as populations not receiving any medical treatment or those receiving a different medication for osteoporosis.

## Abbreviations

ANOVA: Analysis of variance
BMD: Bone mineral density
BV: Balloon volume
CT: Computed tomography
CV: Cement volume
DXA: Dual-energy X-ray absorptiometry
E_2_: Oestradiol
ECLIA: Electrochemiluminescence immunoassays
ELISA: Enzyme-linked immunosorbent assay
FSH: Follicle-stimulating hormone
LH: Luteinizing hormone
MRI: Magnetic resonance imaging
NTX: N-terminal cross-linking telopeptide of type I collagen
OVCF: Osteoporotic vertebral compression fracture
PACS: Picture archiving and communication systems
PKP: Percutaneous kyphoplasty
RCT: Randomized controlled trial
SD: standard deviation
SHBG: Sex hormone binding globulin
TE: Testosterone
VAS: Visual analogue scale
